# Bacterial Contamination of Milk

**Published:** 1904-10-29

**Authors:** 


### Bacterial Contamination of Milk.

A short while ago we published some articles on the organisations which have been established in France for the purpose of reducing the mortality among infants; and we showed how quickly our French neighbours had appreciated the national importance of the work done by the Gouttes de Lait and similar institutions. Every patriotic Frenchman must have welcomed an innovation which promised in some measure to counterbalance the shrinkage which has come about in his country's birth-rate and which points so unmistakably to race suicide. It is not surprising, therefore, that an attempt is now being made to form a central organisation in order to bring existing infant milk depôts under one system and to establish similar depôts wherever there may be need of them. It is probable that the spirit which animates those who have arranged the congress which is now being held for this purpose at Fécamp will quickly spread on this side of the Channel, where the general public is already awakening to the folly and wickedness of allowing every year thousands of babies to die from causes which, with comparatively small expense, might be eliminated by restricting the sale of poisonous milk. And the proposal that municipal authorities should undertake to provide milk guaranteed to be suitable for the feeding of infants has been widely accepted as a feasible and proper solution of the problem. But, while there is no need to question the goodwill of those who are urging, and in some instances securing, the institution of a municipal milk supply, it may be questioned whether their zeal is always according to knowledge. In a very pertinent article in a recent number of the "British Journal of Children's Diseases" it was pointed out that the various milk modifications produced by processes described as sterilising, humanising, etc., are as adequate foods for normal infants yet on their trial. Suitable as they may be in special circumstances and for limited periods there is good reason to doubt their ability to take the place of fresh cow's milk. Indeed, it is definitely stated that cases of disease and deterioration in infants have undoubtedly been traced to these methods of feeding. It is therefore manifest that unless the movement in favour of municipal milk supply is wisely guided it may produce anything but happy results. It may be urged that the proper function of the municipality in this matter is not to establish sterilising depôts, but to trace the sources of contamination and, by removing these, to ensure the supply of a safe and pure milk. In the article to which we have referred the writer advocates the official inspection of farms and shops engaged in the supply and distribution of milk and the control of all such places under municipal license. A contaminated water supply would be met, not by the establishment of a sterilising centre, but by the investigation and removal of the source of the contamination. A similar duty lies upon the municipality in reference to milk. It is in this direction rather than by the organisation of depôts for the distribution of modified milk that the local health authorities can best justify their laudable desire to promote infant well-being. We therefore hope that our countrymen, while following the example of France in procuring innocuous milk supplies for children of tender age, will yet go a step further by putting an end altogether to the indiscriminate sale of grossly contaminated milk. We suspect that it is not the babies only who suffer in consequence of swallowing microbe-laden milk; and in any case efficient regulations for the collection, storage, and distribution of milk, combined with bacterial tests, would strike nearer the fount of the evil, and would in all probability be a better and cheaper means of achieving the desired end than the establishment of infant milk depôts on the plan which has been adopted with such enthusiasm in France.

